# Bilateral Optic Disc Edema in a Patient with Lead Poisoning

**DOI:** 10.18502/jovr.v16i3.9450

**Published:** 2021-07-29

**Authors:** Paolo Pigatto, Gianpaolo Guzzi

**Affiliations:** ^1^Clinical Dermatology, IRCCS Istituto Ortopedico Galeazzi, Milano MI, Italy; ^2^Department of Biomedical, Surgical and Dental Sciences, University of Milan, Milan, Italy; ^3^Italian Association for Metals and Biocompatibility Research – AIRMEB, Milan, Italy

 Dear Editor,

In their important contribution as a “Case Report”, Abri Aghdam et al^[1]^ described a man with bilateral optic disc edema due to lead (Pb) poisoning and complicated by opium addiction.^[1]^ This is an excellent description of papilledema induced by Pb overexposure,^[1]^ which is a rare but serious optic nerve damage attributed to systemic Pb toxicity.^[2,3]^

However, we would like to emphasize the role of whole-blood in diagnosing the Pb poisoning. The patient's serum contained very high levels of Pb, which was 164 µg/dL.^[1]^ In humans, the normal blood Pb level is zero.^[4,5]^

We wonder whether whole-blood Pb concentrations were determined. In our view, the serum is not the primary and proper indicator medium as a biomarker of Pb exposure. In fact, serum alone (with no red blood cells) does not adequately reflect the 2% of the total body burden of the Pb, which is found in the circulating whole-blood.^[4,5]^

In toxicology, the whole-blood Pb concentrations have been used in conjunction with urinary Pb levels as a primary measure of Pb exposure in humans.^[6]^

Toxicological studies suggest that exposure to Pb during developmental periods may lead to long-term visual deficits both in *in vitro* and in animal models.^[7]^

Toxic optic neuropathy may be the unique clinically significant alteration in patients with Pb poisoning.^[2,3]^ Fortunately, Pb poisoning is a rare circumstance not commonly encountered by ophthalmologists.^[8,9,10,11]^ Consistent with this notion, Pb and other toxic metals (i.e., organic mercury, thallium) are to be considered “neurotoxicants”, primarily due to toxic effects on the optic nerve.^[12,13,14]^

With regard to the issue of toxic optic neuropathy, over the past two decades, we have noticed only one case in which papilledema was associated with overexposure to nickel salts. Their interesting case report^[1]^ reminds us that Pb poisoning is a topic of growing interest among ophthalmologists^[15]^ and conveys the fact that the eyes can be injured due to Pb intoxication.

##  Financial Support and Sponsorship

Nil.

##  Conflicts of Interest

There are no conflicts of interest.
